# (*E*,*E*)-1,2-Bis(2,4,6-trimeth­oxy­benzyl­idene)hydrazine

**DOI:** 10.1107/S1600536810033684

**Published:** 2010-08-25

**Authors:** Hoong-Kun Fun, Patcharaporn Jansrisewangwong, Suchada Chantrapromma

**Affiliations:** a X-ray Crystallography Unit, School of Physics, Universiti Sains Malaysia, 11800 USM, Penang, Malaysia; b Department of Chemistry and Center of Excellence for Innovation in Chemistry, Faculty of Science, Prince of Songkla University, Hat-Yai, Songkhla 90112, Thailand; c Crystal Materials Research Unit, Department of Chemistry, Faculty of Science, Prince of Songkla University, Hat-Yai, Songkhla 90112, Thailand

## Abstract

The title mol­ecule, C_20_H_24_N_2_O_6_, lies on an inversion centre. All non-H atoms are essentially coplanar, with an r.m.s. deviation of 0.0415 (1) Å and a maximum deviation of 0.1476 (1) Å for the meth­oxy C atom at the 4-position of the benzene ring. The crystal structure is stabilized by weak C—H⋯N and C—H⋯π inter­actions.

## Related literature

For standard bond-length data, see: Allen *et al.* (1987 ▶). For related structures, see: Jansrisewangwong *et al.* (2010 ▶); Zhao *et al.* (2006 ▶). For background and the biological activity of hydro­zones, see: El-Tabl *et al.* (2008 ▶); Qin *et al.* (2009 ▶); Ramamohan *et al.* (1995 ▶); Rollas & Küçükgüzel (2007 ▶). For the stability of the temperature controller used in the data collection, see Cosier & Glazer, (1986 ▶).
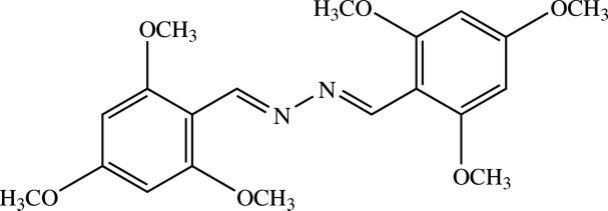


## Experimental

### 

#### Crystal data

C_20_H_24_N_2_O_6_
*M* *_r_* = 388.41
Triclinic, 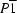

*a* = 7.3851 (2) Å
*b* = 7.4043 (2) Å
*c* = 9.5440 (2) Å
α = 71.412 (1)°
β = 78.095 (1)°
γ = 79.449 (1)°
*V* = 480.13 (2) Å^3^
*Z* = 1
Mo *K*α radiation
μ = 0.10 mm^−1^
*T* = 100 K
0.29 × 0.14 × 0.08 mm

#### Data collection

Bruker APEXII CCD area-detector diffractometer
Absorption correction: multi-scan (*SADABS*; Bruker, 2005 ▶) *T*_min_ = 0.972, *T*_max_ = 0.992
11100 measured reflections
2791 independent reflections
2244 reflections with *I* > 2σ(*I*)
*R*_int_ = 0.025

#### Refinement

*R*[*F* ^2^ > 2σ(*F* ^2^)] = 0.040
*wR*(*F* ^2^) = 0.115
*S* = 1.03
2791 reflections
134 parameters
H atoms treated by a mixture of independent and constrained refinement
Δρ_max_ = 0.42 e Å^−3^
Δρ_min_ = −0.23 e Å^−3^

### 

Data collection: *APEX2* (Bruker, 2005 ▶); cell refinement: *SAINT* (Bruker, 2005 ▶); data reduction: *SAINT*; program(s) used to solve structure: *SHELXTL* (Sheldrick, 2008 ▶); program(s) used to refine structure: *SHELXTL*; molecular graphics: *SHELXTL*; software used to prepare material for publication: *SHELXTL* and *PLATON* (Spek, 2009 ▶).

## Supplementary Material

Crystal structure: contains datablocks global, I. DOI: 10.1107/S1600536810033684/lh5117sup1.cif

Structure factors: contains datablocks I. DOI: 10.1107/S1600536810033684/lh5117Isup2.hkl

Additional supplementary materials:  crystallographic information; 3D view; checkCIF report

## Figures and Tables

**Table 1:** Hydrogen-bond geometry (Å, °) *Cg* is the centroid of the C1–C6 ring.

*D*—H⋯*A*	*D*—H	H⋯*A*	*D*⋯*A*	*D*—H⋯*A*
C10—H10*B*⋯N1^i^	0.96	2.49	3.3876 (15)	155
C8—H8*C*⋯*Cg*^ii^	0.97	2.79	3.6678 (13)	152
C10—H10*C*⋯*Cg*^iii^	0.97	2.63	3.4385 (13)	142

Symmetry codes: (i) 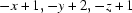; (ii) 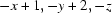; (iii) 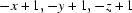.

**Fig. 1.**
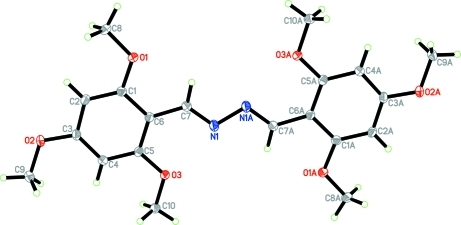
The molecular structure of the title compound, showing 50% probability displacement ellipsoids and the atom-numbering scheme. Atoms with suffix A were generated by symmetry code -x, 2 - y, 1 - z.

**Fig. 2.**
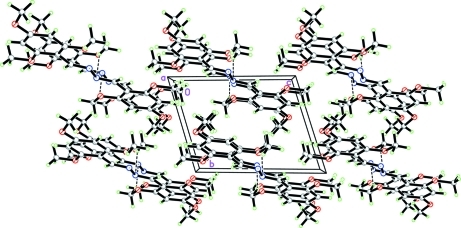
Part of the crystal structure showing weak hydrogen bonds as dashed lines.

## supplementary crystallographic information

### Comment

Hydrazones and their complexes are interesting due to their fluorescence properties (Qin *et al.*, 2009) and various biological activities such as insecticidal, antitumor, antioxidant, antifungal, antibacterial and antiviral properties (El-Tabl *et al.*, 2008; Ramamohan *et al.*, 1995; Rollas & Küçükgüzel, 2007). These interesting properties led us to synthesize the title hydrazone derivative (I) in order to study its antibacterial activity and fluorescence property. Experiments show that (I) does not possess antibacterial activities, however it does exhibit fluorescence with the maximum emission at 410 nm when the compound is excited at 280 nm. Herein the crystal structure of (I) is reported.

The asymmetric unit of (I), (Fig. 1), C_20_H_24_N_2_O_6_, contains one half-molecule and the complete molecule is generated by an inversion centre (symmetry code -x, 2-y, 1-z). The mean plane through the C=N-N=C bridge forms a dihedral angle of 4.96 (9)° with the benzene rings. The methoxy groups attached to atoms C1 and C5 (positions 2 and 6) are approximately coplanar with the benzene ring whereas the one attached to atom C3 (position 4) is slightly twisted with respect to the benzene ring as described by the torsion angles of C8–O1–C1–C2 = 2.86 (15)°, C10–O3–C5–C4 = 3.58 (14)° and C9–O2–C3–C4 = 8.39 (15)°, respectively. The N-N bond length, 1.4117 (18) Å is comparable with 1.419 (3) Å and the C=N-N angle = 110.7 (2)°, is almost similar to 112.2 (2)° observed in (*E*,*E*)-1,2-bis(3,4,5-trimethoxybenzylidene)hydrazine (Zhao *et al.*, 2006). The bond distances have normal values (Allen *et al.*, 1987) and are comparable with related structures (Jansrisewangwong *et al.*, 2010; Zhao *et al.*, 2006). The crystal structure is stabilized by weak C—H···N and C—H···π interactions (Fig. 2).

### Experimental

The title compound was synthesized by mixing a solution (1:2 molar ratio) of hydrazine hydrate (0.097 ml, 2 mmol) and 2,4,6-trimethoxybenzaldehyde (0.785 mg, 4 mmol) in ethanol (20 ml). The resulting solution was refluxed for 5 h, yielding the yellow solid. The resultant solid was filtered off and washed with methanol. Yellow block-shaped single crystals of the title compound suitable for *x*-ray structure determination were recrystalized from acetone by slow evaporation of the solvent at room temperature over several days, mp. 484–486 K.

### Refinement

The H atom attached to C7 was located in a difference map and refined isotropically. The remaining H atoms were positioned geometrically and allowed to ride on their parent atoms, with d(C—H) = 0.93 Å for aromatic and 0.96 Å for CH_3_ atoms. The *U*_iso_ values were constrained to be 1.5*U*_eq_ of the carrier atom for methyl H atoms and 1.2*U*_eq_ for the remaining H atoms. A rotating group model was used for the methyl groups.

### Crystal data

**Table:**

C_20_H_24_N_2_O_6_	*Z* = 1
*M**_r_* = 388.41	*F*(000) = 206
Triclinic, *P*1	*D*_x_ = 1.343 Mg m^−^^3^
Hall symbol: -P 1	Melting point = 484–486 K
*a* = 7.3851 (2) Å	Mo *K*α radiation, λ = 0.71073 Å
*b* = 7.4043 (2) Å	Cell parameters from 2791 reflections
*c* = 9.5440 (2) Å	θ = 2.3–30.0°
α = 71.412 (1)°	µ = 0.10 mm^−^^1^
β = 78.095 (1)°	*T* = 100 K
γ = 79.449 (1)°	Block, yellow
*V* = 480.13 (2) Å^3^	0.29 × 0.14 × 0.08 mm

### Data collection

**Table:**

Bruker APEXII CCD area-detector diffractometer	2791 independent reflections
Radiation source: sealed tube	2244 reflections with *I* > 2σ(*I*)
graphite	*R*_int_ = 0.025
φ and ω scans	θ_max_ = 30.0°, θ_min_ = 2.3°
Absorption correction: multi-scan (*SADABS*; Bruker, 2005)	*h* = −10→10
*T*_min_ = 0.972, *T*_max_ = 0.992	*k* = −10→10
11100 measured reflections	*l* = −13→13

### Refinement

**Table:**

Refinement on *F*^2^	Primary atom site location: structure-invariant direct methods
Least-squares matrix: full	Secondary atom site location: difference Fourier map
*R*[*F*^2^ > 2σ(*F*^2^)] = 0.040	Hydrogen site location: inferred from neighbouring sites
*wR*(*F*^2^) = 0.115	H atoms treated by a mixture of independent and constrained refinement
*S* = 1.03	*w* = 1/[σ^2^(*F*_o_^2^) + (0.0603*P*)^2^ + 0.1087*P*] where *P* = (*F*_o_^2^ + 2*F*_c_^2^)/3
2791 reflections	(Δ/σ)_max_ = 0.001
134 parameters	Δρ_max_ = 0.42 e Å^−^^3^
0 restraints	Δρ_min_ = −0.23 e Å^−^^3^

### Special details

**Table:**

Experimental. The crystal was placed in the cold stream of an Oxford Cryosystems Cobra open-flow nitrogen cryostat (Cosier & Glazer, 1986) operating at 120.0 (1) K.
Geometry. All e.s.d.'s (except the e.s.d. in the dihedral angle between two l.s. planes) are estimated using the full covariance matrix. The cell e.s.d.'s are taken into account individually in the estimation of e.s.d.'s in distances, angles and torsion angles; correlations between e.s.d.'s in cell parameters are only used when they are defined by crystal symmetry. An approximate (isotropic) treatment of cell e.s.d.'s is used for estimating e.s.d.'s involving l.s. planes.
Refinement. Refinement of *F*^2^ against ALL reflections. The weighted *R*-factor *wR* and goodness of fit *S* are based on *F*^2^, conventional *R*-factors *R* are based on *F*, with *F* set to zero for negative *F*^2^. The threshold expression of *F*^2^ > σ(*F*^2^) is used only for calculating *R*-factors(gt) *etc*. and is not relevant to the choice of reflections for refinement. *R*-factors based on *F*^2^ are statistically about twice as large as those based on *F*, and *R*- factors based on ALL data will be even larger.

### Fractional atomic coordinates and isotropic or equivalent isotropic displacement parameters (Å^2^)

**Table:**

	*x*	*y*	*z*	*U*_iso_*/*U*_eq_	
O1	0.20858 (11)	0.87907 (11)	0.08848 (8)	0.01756 (18)	
O2	0.82788 (11)	0.57545 (11)	0.12544 (8)	0.01906 (19)	
O3	0.44058 (10)	0.78425 (11)	0.53298 (8)	0.01517 (17)	
N1	0.09376 (12)	0.95751 (13)	0.49155 (10)	0.0163 (2)	
C1	0.35619 (14)	0.81164 (14)	0.16438 (11)	0.0142 (2)	
C2	0.52565 (15)	0.72803 (15)	0.10450 (11)	0.0158 (2)	
H2A	0.5443	0.7178	0.0078	0.019*	
C3	0.66759 (14)	0.65957 (15)	0.19168 (11)	0.0148 (2)	
C4	0.64415 (14)	0.67699 (14)	0.33540 (11)	0.0143 (2)	
H4A	0.7406	0.6318	0.3917	0.017*	
C5	0.47341 (14)	0.76345 (14)	0.39383 (10)	0.0133 (2)	
C6	0.32381 (14)	0.83151 (14)	0.31125 (11)	0.0134 (2)	
C7	0.14037 (15)	0.91807 (15)	0.36584 (11)	0.0147 (2)	
C8	0.22823 (17)	0.85390 (17)	−0.05710 (12)	0.0205 (2)	
H8A	0.1127	0.9008	−0.0954	0.031*	
H8B	0.2601	0.7199	−0.0499	0.031*	
H8C	0.3251	0.9241	−0.1234	0.031*	
C9	0.96932 (16)	0.47848 (17)	0.21582 (12)	0.0212 (2)	
H9A	1.0697	0.4173	0.1585	0.032*	
H9B	0.9173	0.3834	0.3022	0.032*	
H9C	1.0157	0.5698	0.2473	0.032*	
C10	0.59168 (15)	0.72427 (16)	0.61730 (11)	0.0167 (2)	
H10A	0.5516	0.7495	0.7123	0.025*	
H10B	0.6945	0.7939	0.5631	0.025*	
H10C	0.6302	0.5892	0.6330	0.025*	
H7	0.0407 (19)	0.9430 (19)	0.3064 (15)	0.022 (3)*	

### Atomic displacement parameters (Å^2^)

**Table:**

	*U*^11^	*U*^22^	*U*^33^	*U*^12^	*U*^13^	*U*^23^
O1	0.0168 (4)	0.0235 (4)	0.0134 (3)	0.0022 (3)	−0.0047 (3)	−0.0080 (3)
O2	0.0149 (4)	0.0236 (4)	0.0163 (4)	0.0049 (3)	0.0002 (3)	−0.0082 (3)
O3	0.0145 (4)	0.0196 (4)	0.0124 (3)	0.0013 (3)	−0.0034 (3)	−0.0073 (3)
N1	0.0125 (4)	0.0186 (4)	0.0173 (4)	−0.0003 (3)	−0.0004 (3)	−0.0069 (3)
C1	0.0156 (5)	0.0131 (4)	0.0133 (4)	−0.0010 (4)	−0.0031 (4)	−0.0032 (4)
C2	0.0175 (5)	0.0173 (5)	0.0122 (4)	−0.0009 (4)	−0.0001 (4)	−0.0058 (4)
C3	0.0134 (5)	0.0145 (5)	0.0150 (4)	−0.0007 (4)	0.0012 (4)	−0.0049 (4)
C4	0.0133 (5)	0.0149 (5)	0.0144 (4)	−0.0008 (4)	−0.0028 (4)	−0.0043 (4)
C5	0.0153 (5)	0.0120 (4)	0.0123 (4)	−0.0028 (4)	−0.0003 (4)	−0.0040 (3)
C6	0.0140 (5)	0.0134 (4)	0.0126 (4)	−0.0010 (4)	−0.0014 (3)	−0.0046 (4)
C7	0.0137 (5)	0.0151 (5)	0.0151 (4)	−0.0006 (4)	−0.0027 (4)	−0.0049 (4)
C8	0.0240 (6)	0.0251 (6)	0.0143 (5)	0.0020 (4)	−0.0061 (4)	−0.0093 (4)
C9	0.0159 (5)	0.0242 (5)	0.0198 (5)	0.0043 (4)	−0.0014 (4)	−0.0061 (4)
C10	0.0157 (5)	0.0207 (5)	0.0157 (4)	−0.0010 (4)	−0.0051 (4)	−0.0071 (4)

### Geometric parameters (Å, °)

**Table:**

O1—C1	1.3632 (12)	C4—H4A	0.9300
O1—C8	1.4347 (12)	C5—C6	1.4135 (14)
O2—C3	1.3642 (12)	C6—C7	1.4564 (14)
O2—C9	1.4328 (13)	C7—H7	0.976 (14)
O3—C5	1.3528 (11)	C8—H8A	0.9600
O3—C10	1.4322 (12)	C8—H8B	0.9600
N1—C7	1.2882 (13)	C8—H8C	0.9600
N1—N1^i^	1.4117 (18)	C9—H9A	0.9600
C1—C2	1.3866 (14)	C9—H9B	0.9600
C1—C6	1.4226 (13)	C9—H9C	0.9600
C2—C3	1.3944 (15)	C10—H10A	0.9600
C2—H2A	0.9300	C10—H10B	0.9600
C3—C4	1.3909 (13)	C10—H10C	0.9600
C4—C5	1.3974 (14)		
			
C1—O1—C8	118.01 (8)	N1—C7—C6	125.41 (9)
C3—O2—C9	117.79 (8)	N1—C7—H7	115.8 (8)
C5—O3—C10	117.61 (8)	C6—C7—H7	118.7 (8)
C7—N1—N1^i^	110.66 (11)	O1—C8—H8A	109.5
O1—C1—C2	122.94 (9)	O1—C8—H8B	109.5
O1—C1—C6	115.10 (9)	H8A—C8—H8B	109.5
C2—C1—C6	121.95 (9)	O1—C8—H8C	109.5
C1—C2—C3	118.90 (9)	H8A—C8—H8C	109.5
C1—C2—H2A	120.5	H8B—C8—H8C	109.5
C3—C2—H2A	120.5	O2—C9—H9A	109.5
O2—C3—C4	123.44 (9)	O2—C9—H9B	109.5
O2—C3—C2	115.02 (9)	H9A—C9—H9B	109.5
C4—C3—C2	121.55 (9)	O2—C9—H9C	109.5
C3—C4—C5	118.99 (9)	H9A—C9—H9C	109.5
C3—C4—H4A	120.5	H9B—C9—H9C	109.5
C5—C4—H4A	120.5	O3—C10—H10A	109.5
O3—C5—C4	122.15 (9)	O3—C10—H10B	109.5
O3—C5—C6	116.17 (9)	H10A—C10—H10B	109.5
C4—C5—C6	121.67 (9)	O3—C10—H10C	109.5
C5—C6—C1	116.93 (9)	H10A—C10—H10C	109.5
C5—C6—C7	124.92 (9)	H10B—C10—H10C	109.5
C1—C6—C7	118.15 (9)		
			
C8—O1—C1—C2	2.86 (15)	C3—C4—C5—C6	0.69 (15)
C8—O1—C1—C6	−176.61 (9)	O3—C5—C6—C1	179.70 (8)
O1—C1—C2—C3	−178.70 (9)	C4—C5—C6—C1	−1.27 (15)
C6—C1—C2—C3	0.74 (16)	O3—C5—C6—C7	−0.86 (15)
C9—O2—C3—C4	8.39 (15)	C4—C5—C6—C7	178.16 (9)
C9—O2—C3—C2	−171.46 (9)	O1—C1—C6—C5	−179.97 (8)
C1—C2—C3—O2	178.49 (9)	C2—C1—C6—C5	0.55 (15)
C1—C2—C3—C4	−1.37 (16)	O1—C1—C6—C7	0.55 (14)
O2—C3—C4—C5	−179.18 (9)	C2—C1—C6—C7	−178.93 (9)
C2—C3—C4—C5	0.67 (16)	N1^i^—N1—C7—C6	179.28 (10)
C10—O3—C5—C4	3.58 (14)	C5—C6—C7—N1	5.52 (17)
C10—O3—C5—C6	−177.40 (8)	C1—C6—C7—N1	−175.05 (10)
C3—C4—C5—O3	179.66 (9)		

Symmetry codes: (i) −*x*, −*y*+2, −*z*+1.

### Hydrogen-bond geometry (Å, °)

**Table:**

Cg is the centroid of the C1–C6 ring.

**Table:**

*D*—H···*A*	*D*—H	H···*A*	*D*···*A*	*D*—H···*A*
C7—H7···O1	0.977 (14)	2.332 (14)	2.6886 (12)	100.6 (10)
C10—H10B···N1^ii^	0.96	2.49	3.3876 (15)	155
C8—H8C···Cg^iii^	0.97	2.79	3.6678 (13)	152
C10—H10C···Cg^iv^	0.97	2.63	3.4385 (13)	142

Symmetry codes:  (ii) −*x*+1, −*y*+2, −*z*+1; (iii) −*x*+1, −*y*+2, −*z*; (iv) −*x*+1, −*y*+1, −*z*+1.
